# Effect of L-carnitine on sperm quality during liquid storage of boar semen

**DOI:** 10.5713/ajas.19.0455

**Published:** 2019-12-24

**Authors:** Kang Yang, Na Wang, Hai-Tao Guo, Jing-Ran Wang, Huan-Huan Sun, Liang-Zhen Sun, Shun-Li Yue, Jia-Bo Zhou

**Affiliations:** 1 Department of Biotechnology, College of Life Science Northeast Agricultural University, Harbin 150030, China; 2 Key Laboratory of Animal Cellular and Genetics Engineering of Heilongjiang Province, Northeast Agricultural University, Harbin 150030, China

**Keywords:** Boar, Sperm, L-carnitine, Liquid Storage, Antioxidant Capacity

## Abstract

**Objective:**

This study was conducted to investigate the effect of L-carnitine on the pig semen characteristics during storage.

**Methods:**

Spermatozoa samples were examined for spermatozoa quality and then randomly divided into 5 groups: 0 (control), 12.5, 25, 50, and 100 mM L-carnitine. Sperm motility, plasma membrane integrity and antioxidant parameters (total reactive oxygen species, total antioxidant capacity, and malondialdehyde) were evaluated after 0, 3, 5, and 10 day cooled-storage at 17°C. Moreover, ATP content, mitochondria activity as well as sperm-binding and *in vitro* fertilizing ability of preserved boar sperm were also investigated.

**Results:**

Supplementation with 50 mM L-carnitine could effectively maintain boar sperm quality parameters such as sperm motility and membrane integrity. Besides, we found that L-carnitine had positive effects on boar sperm quality mainly through improving antioxidant capacities and enhancing ATP content and mitochondria activity. Interestingly, by assessing the effect of L-carnitine on sperm fertility and developmental potential, we discovered that the extender containing L-carnitine could improve sperm quality and increase the number of sperms bounding to zona pellucida, without improving *in vitro* fertility and development potential.

**Conclusion:**

These findings suggested that the proper addition of L-carnitine to the semen extender improved boar sperm quality during liquid storage at 17°C.

## INTRODUCTION

Artificial insemination and semen liquid storage have significantly improved the breeding potential of male animals. However, current liquid preservation techniques commonly result in compromised semen quality, and development potential of sperm is affected by the process [1]. Although special attention has already been devoted to prolonging the viability and fertilizing potential of stored liquid semen, limited improvements have been achieved [2]. A specific problem in the preservation of boar semen is reactive oxygen species (ROS) accumulation which can cause oxidative injury of sperm, resulting in damage of plasma membrane during the long-time liquid preservation [3,4]. Therefore, it’s necessary to find an inexpensive and efficient additive to improve the quality of sperm during the storage.

Carnitine is a quaternary ammonium compound involved in metabolism in most mammals, plants and microorganisms [5,6]. It plays an important role in fatty acid metabolism by transporting fatty acids across the mitochondrial membrane. Carnitine forms a long chain acetylcarnitine ester transported by carnitine palmitoyltransferase I and carnitine palmitoyltransferase II located in the outer and inner mitochondrial membranes allowing fatty acid across the mitochondrial membranes to complete the β-oxidation pathway [7]. Carnitine is mainly synthesized in the liver and at high concentrations in mammalian epididymides and sperms. Epididymal epithelium and sperms obtain energy from the epididymal fluid by carnitine. It has been shown that high concentration of carnitine increases the motility of sperm in epididymal fluid. Besides carnitine is one of effective antioxidants. It reduces the availability of lipids for peroxidation by transporting fatty acids into the mitochondria for β-oxidation to generate ATP. It has also been reported that carnitine protects the activity of pyruvate dehydrogenase which plays a crucial role in mitochondrial respiration by trapping excess mitochondrial acetyl-CoA as acetyl-L-carnitine. The importance of carnitine in sperm quality *in vivo* is well recognized [8]. Previous data have indicated that dietary L-carnitine (LC) supplement could improve semen quality in human [8], chicken [9], and Pietrain boar [10] sperm. Moreover, several studies reported that extender supplemented with LC has positive effects on semen quality in some species, such as stallion [11], rooster [12], rabbit [13], and bull [14] while there are few reports about the effect of LC on liquid preserved boar sperm as well as its fertility potential. Therefore, the aim of our study was to evaluate the effect of LC supplement to Androhep semen extender on motility and membrane integrity of boar sperm. Furthermore, antioxidant parameters (total ROS [t-ROS], total antioxidant capacity [T-AOC], and malondialdehyde [MDA]) of boar sperm as well as ATP content, mitochondria activity, *in vitro* fertilizing ability and embryo developmental potential of preserved boar sperm were also investigated.

## MATERIALS AND METHODS

All experimental procedures involving animals were conducted in accordance with the Guide for Care and Use of Animals in Research and approved by the Institutional Animal Care and Use Committee of Northeast Agricultural University. IACUC (Approval number: 20180912). Unless noted, all chemicals and media used in this study were purchased from Sigma Chemical Co (St. Louis, MO, USA).

### Semen collection

Ejaculates were collected by gloved-hand method from four Yorkshire male pigs (1.5 to 2.5 years age). The boars were allowed at least 3 days of sexual rest between collections. The semen was transported to the laboratory at 37°C to 38°C within 30 min. Only ejaculates containing greater than 70% sperm total motility and less than 15% morphologically abnormal spermatozoa were used in the study. After the evaluation of fresh semen quality, all fresh semen samples were pooled and divided into several equal fractions according to experimental design.

### Semen processing of liquid storage

The Androhep medium containing glucose (26 g/L), sodium citrate (8 g/L), disodium salt dihydrate (2.4 g/L), sodium bicarbonate (1.2 g/L), hepes (9 g/L), bovine serum albumin (2.5 g/L), penethamate (1,000,000 IU/L) and streptomycin (1 g/L) with an osmolarity of approximately 310 mOsm/kg, was utilized as extender throughout this study. The spermatozoa were diluted to 1×10^7^ spermatozoa/mL in Androhep supplemented with different concentrations of LC 0 (control), 12.5, 25, 50, and 100 mM. The diluted semen samples were dispersed into 100 mL plastic bottles and equilibrated for 2 h at room temperature prior to storage at 17°C (Thermo-box, FYL-12MC-B4, China).

### Assessment of sperm motility

Total motility sperm was evaluated using the CASA system (Sperm Class Analyzer, Microptic SL, Barcelona, Spain). Semen samples were placed in a chamber and examined at 38.5°C under a phase-contrast microscopy system coupled to a video camera adapted to the Video Test Sperm system.

### Assessment of sperm membrane integrity

Sperm membrane integrity was evaluated by the hypo-osmotic swelling test. Briefly, 10 μL of sperm sample were added to 200 μL of pre-warmed hypotonic solution (9.0 g of fructose and 4.9 g of sodium citrate per litre of distilled water with an osmolality of 150 mOsm) and mixed thoroughly. After a 45-min incubation at 38.5°C, 200 μL 2% glutaraldehyde were added. After incubation, 15 μL of the mixture were spread with a cover slip on a warm slide. The tail coiling rate of the spermatozoa was examined using a phase-contrast microscope (Leica, Wetzlar, Germany) at a magnification of 400×. Counting of cells was conducted on individual spermatozoa in five to six different medium squares until 200 sperm had been counted. There were three technical replications for all groups.

### Lipid peroxidation measurement

The MDA concentrations in sperm, as an indicator of lipid peroxidation, were measured using commercially available kit (Jiancheng Bioengineering Institute, Jiangsu, China) based on the reaction with 2-thiobarbituricacid and monitored by the change of absorbance at 532 nm with a spectrophotometer. The MDA content in spermatozoa was expressed as nmol/10^8^ sperm cells [15].

### Total antioxidant capacity measurement

The T-AOC was assessed using a total antioxidant capacity assay kit (Jiancheng Bioengineering Institute, Jiangsu, China), according to the manufacturer’s instructions. The T-AOC assay was measured on a spectrophotometer at 520 nm.

### Total reactive oxygen species measurement

Level of sperm intracellular tROS was measured using the method described by Fu et al [16]. In brief, the sperm suspension (5×10^5^ cells/mL spermatozoa) was incubated with 2′, 7′-dichlorofluorescein diacetate (DCFH-DA; Jiancheng Bioengineering Institute, Jiangsu, China) at 37°C for 60 min in the dark, and then the labelled spermatozoa were analyzed by fluorescence microscope. The fluorescence was excited at the wave length of 485 nm and the corresponding emission wave length was 520 nm. ROS fluorescence intensity was examined under a fluorescence microscope (Leica, Germany) with an analysis software system.

### Determination of ATP content

ATP content was assessed using an ATP content assay kit (Jiancheng Bioengineering Institute, Jiangsu, China), according to the manufacturer’s instructions. The ATP content assay was measured on a spectrophotometer (Eppendorf, Hamburg, Germany) at 636 nm.

### Detection of mitochondrial activity

Sperm mitochondrial staining was determined using Mito Tracker deep red (MTDR) fluorescence (Yeasen, Shanghai, China). Evaluations were performed by flow cytometry (BD Biosciences, Franklin Lakes, NJ, USA). In brief, the sperm suspension was incubated with MTDR (final concentration of 100 nM) at 37°C for 30 min in the dark, then the labeled spermatozoa were analyzed by flow cytometry (BD Biosciences, USA). The FL1 signals were detected by a 644 nm band-pass filter. All data were analyzed by FlowJo v7.6.1 software (TreeStar, Inc., Ashland, OR, USA).

### Sperm-binding and *in vitro* fertilization assay

Sperm-binding assay was conducted as described previously [17]. Briefly, approximately 30 to 35 matured oocytes with a first polar body were transferred into a 50-μL droplet of *in vitro* fertilization (IVF) medium that was covered with mineral oil and had been equilibrated at 38.5°C in 5% CO_2_ in air. The preserved spermatozoa were resuspended in the fertilization medium to a concentration of 1×10^6^ cells/mL and capacitated by an additional 1 h of incubation at 38.5°C. Sperm samples were added to the fertilization droplets that contained the oocytes, which gave a final sperm concentration of 0.25×10^6^ cells/mL. Oocytes were then coincubated with sperm for 1 h. For sperm binding assay, the oocytes were fixed with acetic acid/ethanol (1:3) for 5 h. After three washings, the oocytes were stained with Hoechst33342. Bound sperm were observed by confocal microscopy. For IVF, oocytes were then coincubated with sperm for 4 to 6 h. After fertilization, oocytes were washed 3 times and cultured with 500 mL of porcine zygote medium in 4-well dishes at 38.5°C, 5% CO_2_. The cleavage rate was determined at day 2, and the blastocyst rate was analyzed at day 6.

### Statistical analysis

Three replicates of semen were used for *in vitro* evaluation of sperm parameters. All percentage data were analyzed by Chi square test using SPSS 23.0 statistical software (SPSS, Inc., Chicago, IL, USA 2007). Differential staining data were analyzed by the Student’s t test. Data were expressed as the mean ±standard error of the mean, and the p value of less than 0.05 (p<0.05) was considered significant.

## RESULTS

### Effect of L-carnitine on sperm quality during liquid semen storage

The effects of LC on boar sperm motility during preservation at 17°C are shown in Table 1. No significant differences were observed among treatments for up to 3 days. However, at day 5, the total motility of spermatozoa in the groups incubated with 25, 50, and 100 mM LC respectively, was significantly higher than those of 12.5 mM LC and the control (p<0.05). On the day 10 the sperm total motility of LC treated groups were higher than that of the control (p<0.05).

Table 2 shows there were no significant differences in membrane integrity among different LC treatment groups compared with the control at the beginning of storage (at least 3 days). At day 5, higher membrane integrity was observed in LC treated groups compared to the control, especially in the 50 mM LC group. At day 10, the sperm membrane integrity incubated with 50 mM LC was significantly higher than the control (p<0.05).

The results demonstrated the supplement with 50 mM LC has a positive effect on boar sperm motility and membrane integrity. Thus, we used 50 mM LC for the subsequent experiments.

### Effect of L-carnitine on sperm antioxidant capacity

As shown in Table 3, the addition of 50 mM LC did not induce any significant effects on antioxidant parameters (t-ROS, T-AOC, and MDA) of boar sperm compared with the control at the beginning of storage (at least 3 days). After 10 days of preservation, the MDA content and t-ROS levels of sperm increased while LC reduced MDA and ROS accumulation. On the other hand, LC supplementation notably reversed the T-AOC activity of sperm declining induced by storage (p<0.05). Given above results, we concluded that 50 mM LC treatment could maintain the AOC of boar sperm for at least ten days.

### Effect of L-carnitine on sperm ATP levels and mitochondria activity

Results of sperm ATP levels are presented in Table 4. The addition of 50 mM LC did not have any effects on ATP content at 0 day. After 3 days of incubation, ATP content was significantly (p<0.05) higher in LC treated group than the control. During the following preserved periods the ATP levels were significantly (p<0.05) higher in treated groups than control. Moreover, the supplement of LC had a positive effect on the mitochondrial activity rate. Mitochondrial activity was significantly (p<0.05) higher in LC group than the control after 5 days of preservation (Table 4).

### Effect of L-carnitine supplement on fertility and development potential of boar sperm

As shown in Table 5, the addition of LC induced more sperms binding to zona pellucida (ZP) compared with the control (p<0.05). The penetration rate of IVF in LC treated group was higher than that of the control (p<0.05) while the total efficiency and monospermy rates were not affected by LC. Similarly, LC treatment did not exert any effects on the cleavage and blastocyst rates when the sperm was stored for 5 days (Table 5).

## DISCUSSION

Liquid storage of mammalian semen can cause several time-dependent structural and biochemical damages to sperm and lead to the decreased fertilizing ability of preserved sperm. In the present study, we observed a reduction of sperm total motility and plasma membrane integrity in all groups in liquid storage as time went on. However, this reduction in groups containing LC was less than the control. Furthermore, supplement of Androhep with 50 mM LC produced higher sperm motility and plasma membrane integrity compared to other groups during the period of liquid storage.

The use of exogenous antioxidants is a key strategy to alleviate time-dependent structural and biochemical damage of sperm caused by inappropriate formation of ROS in the liquid preserved boar semen [18]. Antioxidant properties of LC include the scavenging of free radicals, destruction of hydrogen peroxide and metal chelation [19] as well as inhibition of xanthine oxidase activity [20]. Among the antioxidant properties of LC, the reduction of lipid peroxidation is most widely reported and is routinely exploited in clinical settings such as reducing the severity of damage caused by ischemia-reperfusion-induced lipid peroxidation following organ surgery [21]. In this study, the MDA concentrations and t-ROS levels in the group with 50 mM LC were lower than those of the control in the meanwhile the supplementation of LC significantly increased the T-AOC activity of preserved sperm. This suggested that LC treatment could maintain the AOC of boar sperm for at least ten days. Those results of this study agree with previous reports of the beneficial effects of LC on spermatozoa of other species such as human [6], rooster [9], rat [22], cat [23], and other cell types [24–26].

Moreover, our results showed that supplement with LC could increase sperm ATP content and mitochondrial activity. This may be attributed to the role of LC in metabolism [6]. Because of the effect of facilitating fatty acids transport across the inner membrane of mitochondria for β-oxidation [27], LC plays a crucial role in mitochondrial ATP production, thus providing a better supply of energy to increase sperm motility. LC traps extra mitochondrial acetyl-CoA as acetyl-L-carnitine, which decreases the acetyl-CoA: free CoA ratio, enhancing the ability of the pyruvate dehydrogenase and promoting the citric acid cycle, thus increasing the production of ATP and mitochondrial activity. Moreover, LC is powerful osmolyte [20]. In fact, supplement of extender with LC leads to partial removal of NaCl from diluent to maintain isotonicity while NaCl in the solution accelerates the depletion of ATP through the activation of Na-ATPase pumps. The ATP-dependent Na+/K+ pump can consume up to 20% of the ATP produced by a cell in an attempt to maintain homeostasis [28]. Therefore, the motility of sperm is increased by LC through saving and promoting mitochondrial ATP production.

To assess whether LC treatment has positive effects on improving sperm fertility and developmental potential, the number of sperm binding to oocytes, total fertilization efficiency, cleavage rate, and blastocyst rate were performed using boar sperm which was preserved for 5 days in Androhep with or without 50 mM LC. The ZP binding ability of sperm is crucial for sperm because sperm-oocyte interactions determine successful fertilization. Our results indicate that the sperm binding ability and the penetration rate of IVF could be improved by LC, while the total efficiency of the monospermy rates, cleavage and blastocyst rates were indeed unaffected. Therefore, the supplement of the extender with LC could improve sperm quality and increase the number of sperms bounding to ZP, without improving *in vitro* fertility and development potential.

In conclusion, the supplement of semen extender with LC significantly increased boar sperm total motility and membrane integrity. Moreover, we found that the positive effects of LC on boar sperm quality were mainly due to scavenging ROS, enhancing boar sperm antioxidant properties and increasing sperm ATP levels and mitochondria activity. Despite the supplementation of LC improving sperm quality, the percentage of normospermic penetration after IVF and the devolvement potential of embryos were not influenced.

## Figures and Tables

**Table 1:** Effect of L-carnitine on boar sperm motility during preservation

Periods of preservation (d)	Total motility (%)				

	0 mM LC	12.5 mM LC	25 mM LC	50 mM LC	100 mM LC
0	86.07±0.71^a^	87.13±0.95^a^	86.18±1.06^a^	83.60±1.65^a^	86.06±0.89^a^
3	77.97±1.03^a^	73.51±1.84^a^	76.30±1.60^a^	78.40±0.55^a^	73.26±1.09^a^
5	42.83±0.55^a^	48.36±1.41^a^	52.52±0.78^b^	55.40±0.56^c^	50.89±0.84^b^
10	20.47±0.70^a^	25.92±1.60^b^	30.05±0.34^b^	34.07±0.45^c^	24.06±0.62^b^

Data are represented as the mean±standard error of the mean (n = 3).

LC, L-carnitine.

a–c Different superscript within the same row indicates significant differences at p<0.05.

**Table 2:** Effects of L-carnitine on boar sperm plasma membrane integrity during preservation

Periods of preservation (d)	Plasma membrane integrity (%)				

	0 mM LC	12.5 mM LC	25 mM LC	50 mM LC	100 mM LC
0	80.78±0.94^a^	82.09±1.47^a^	81.80±0.92^a^	80.15±0.10^a^	81.37±0.62^a^
3	54.58±0.46^a^	56.03±0.50^a^	55.45±0.40^a^	56.46±0.37^a^	53.89±0.54^a^
5	33.05±0.29^a^	36.95±0.36^b^	36.34±0.49^bc^	41.28±0.57^c^	35.92±0.30^b^
10	12.77±0.52^a^	16.95±0.05^b^	18.35±0.68^bc^	22.98±0.75^c^	17.55±0.13^b^

Data are represented as mean±standard error of the mean (n = 3).

LC, L-carnitine.

a–c Different superscript within the same row indicates significant differences at p<0.05.

**Table 3:** Effects of L-carnitine on t-ROS levels, T-AOC activity, and MDA content of boar sperm

Periods of preservation (d)	t-ROS levels (ɛ)		T-AOC activity (U/mL)		MDA (nmol/10^8^ sperm)	
		
	0 mM LC	50 mM LC	0 mM LC	50 mM LC	0 mM LC	50 mM LC
0	18.86±0.81^a^	18.90±0.90^a^	7.81±0.09^a^	7.42±0.08^a^	0.55±0.09^a^	0.61±0.15^a^
3	41.24±1.05^a^	40.63±0.89^a^	4.58±0.14^a^	4.55±0.10^a^	0.62±0.36^a^	0.66±0.49^a^
5	57.07±1.42^a^	47.04±1.06^b^	3.10±0.11^a^	4.17±0.12^b^	1.57±0.12^a^	1.11±0.08^b^
10	72.77±2.95^a^	61.52±0.95^b^	2.25±0.08^a^	3.29±0.12^b^	2.64±0.39^a^	1.48±0.05^b^

Data are represented as mean±standard error of the mean (n = 3).

LC, L-carnitine; t-ROS, total reactive oxygen species; T-AOC, total antioxidant capacity; MDA, malondialdehyde.

a,b Different superscript between two LC level at the same period of preservation indicates significant differences at p<0.05.

**Table 4:** Effects of L-carnitine on boar sperm ATP content and mitochondrial activity during preservation

Periods of preservation (d)	ATP content (nmol/10^8^ sperm)		Mitochondrial activity	
	
	0 mM LC	50 mM LC	0 mM LC	50 mM LC
0	13.35±0.33^a^	13.25±0.40^a^	83.65±0.96^a^	81.93±1.18^a^
3	8.99±0.42^a^	10.99±0.36^b^	66.51±0.70^a^	69.60±0.82^a^
5	7.26±0.20^a^	10.30±0.19^b^	38.14±1.28^a^	57.21±1.93^b^
10	4.85±0.28^a^	8.55±0.26^b^	13.72±0.99^a^	21.65±1.29^b^

Data are represented as mean±standard error of the mean (n = 3).

LC, L-carnitine.

a,b Different superscript between two LC level at the same period of preservation indicates significant differences at p<0.05.

**Table 5:** Effects of L-carnitine on boar sperm fertility and developmental potential

Treatment	Oocytes examined (n)	Sperm bound to ZP (n)	Penetration rate (%)	Monospermy rate (%)	Total efficiency of IVF (%)	Cleavage rate (%)	Blastocyst rate (%)
Control	432	78.81±0.65^a^	42.26±1.16^a^	57.55± 0.52^a^	26.72±0.31^a^	64.28±1.09^a^	27.37±0.49^a^
50 mM L-carnitine	570	91.20±1.03^b^	54.91±1.08^b^	58.17±0.80^a^	30.85±0.94^a^	66.03±1.51^a^	29.55±0.82^a^

Sperm bound to ZP means sperm binding to the surface of the zona pellucida (n = number of bound spermatozoa per oocyte). The penetration rate means number of oocytes fertilized vs number of inseminated oocytes. The monospermy rate means number of oocytes containing one sperm head-male pronucleus vs number of penetrated oocytes. The total efficiency of fertilization means number of monospermic oocytes vs number of inseminated oocytes. The cleavage rate means the number of 2-cell embryo vs number of zygotes. The blastocyst rate means the number of blastocysts vs number of zygotes.

Values are expressed as the mean±standard error of the mean (n = 6).

ZP, zona pellucida; IVF, *in vitro* fertilization.

a,b Different letters indicate significant difference at p<0.05 in column.
